# Knowing and Unknowing Purchases of Undeclared Healthcare Goods and Services: The Role of Vertical and Horizontal Trust

**DOI:** 10.3390/ijerph182111561

**Published:** 2021-11-03

**Authors:** Ioana Alexandra Horodnic, Colin C. Williams, Alexandru Maxim, Iuliana Claudia Stoian, Oana Carmen Țugulea, Adrian V. Horodnic

**Affiliations:** 1 Faculty of Economics and Business Administration, Alexandru Ioan Cuza University of Iași, 700505 Iași, Romania; maxim.alexandru@uaic.ro (A.M.); iuliana.bobalca@uaic.ro (I.C.S.); ciobanu.oana@uaic.ro (O.C.Ț.); 2 Management School, University of Sheffield, Sheffield S10 2TN, UK; c.c.williams@sheffield.ac.uk; 3 Faculty of Medicine, Grigore T. Popa University of Medicine and Pharmacy, 700115 Iași, Romania; adi.horodnic@gmail.com

**Keywords:** undeclared healthcare goods and services, informal economy, horizontal trust, vertical trust, consumer behaviour

## Abstract

Although major advances have been made in relation to explaining the supply side of the informal economy, this is not the case for the demand-side of the informal economy. This study analyses for the first time the purchasers of undeclared goods and services in the healthcare sector. To evaluate the purchase of undeclared healthcare goods and services, logistic regression analysis and robustness tests are used on 3048 interviews in Cyprus, Greece, Italy and Malta. The finding is that an important share of the purchasers make this type of purchase unknowingly. However, no difference in terms of socio-economics characteristics of those who knowingly and those who unknowingly made purchases of undeclared healthcare goods and services was identified. Meanwhile a significant influence of trust (in government and in other citizens) has been identified in relation to those who made these purchases knowingly. As such, policy measures aimed at decreasing unknowing purchases and at nurturing trust are discussed in the concluding section.

## 1. Introduction

Over the past few decades, many studies have sought to understand and explain participation in the informal economy. Although major advances have been made, this has mostly been in relation to explaining the supply side of the informal economy or the providers of informal goods and services [1,2]. Despite some 60% of the global workforce having their main employment in the informal economy [3,4], displaying that the informal economy is a large segment of global production and consumption, few have analysed who makes purchases of undeclared goods and services (i.e., the demand side of the informal economy) and why they do so. There are only a few notable exceptions [5,6,7,8,9,10]. Even fewer studies have evaluated the motives driving individuals to make purchases from the informal economy, including whether individuals are sometimes unaware that they are purchasing from the informal economy [11,12]. Although corruption and informal practices in the healthcare sector are well documented, no previous study has investigated who make purchases of undeclared healthcare goods and services and why they do so. Instead, in the healthcare sector, the focus has been upon the supply side, focusing upon explaining, for example, corruption in procurements [13,14] or informal payments to healthcare practitioners [15,16,17,18,19,20]. As such, studies have not evaluated the purchaser of undeclared healthcare goods and services and sought to explain whether they do so knowingly or not, and from whom they make such purchases.

To fill this significant gap, this study analyses for the first time the purchasers of undeclared goods and services in the healthcare sector. The paper‘s novelty is twofold. Firstly, unlike the previous studies on the demand side of the informal economy, this study accounts for both categories of motives that can drive consumers to informal economy, namely whether they do so knowingly or unknowingly. Secondly, this is the first paper that seeks to evaluate the influence of both types of trust (i.e., vertical or the trust that citizens have in their government and horizontal trust or the trust that citizens have in their fellow citizens) in purchasing healthcare goods and services. These issues have been found to be significant in relation to the supply side of informal economy (i.e., those providing the undeclared goods and services) and we here propose to test their validity in terms of the demand side of the informal economy.

Indeed, when explaining those providing the undeclared goods and services, two main groups have been identified, namely those who use this strategy for survival, having no other choice (“exclusion” or “marginalization” thesis) [21,22,23] and those who choose to engage in informal economy (“exit” or “reinforcement” thesis) for various reasons ranging from avoiding burdensome regulations to meeting social ends [24,25,26,27]. When analysing the supply side of the informal economy in the European Union member states, the finding is that there is indeed a dual informal labour market and that the socio-economic characteristics of the exclusion- and exit-driven informal workers differ. The exclusion-driven “lower-tier” of the informal economy is more likely to be composed of the unemployed and workers in East-Central Europe, whilst the exit-driven “upper tier” is more likely to be formed by those with few financial difficulties and workers in Nordic nations [28]. In relation to the demand-side of the informal economy, no previous study has investigated the socio-demographic characteristics of those who purchase undeclared goods and services (in general or in healthcare sector) and whether a similar dual market exists between those who purchase on a knowing basis against those who make such purchases unknowingly. However, previous literature has identified that there are some socio-demographic variations in relation to various knowing motivations, such as seeking a lower price, seeking a faster or better service, having no choice due to the lack of availability on the formal market, or seeking social ends [5,7,8,9,10,11,12]. As such, we propose the following hypothesis to be tested:

**Hypothesis 1** **(H1).** *The socio-demographic profile of the purchaser varies according to the different drivers that determine the purchase of healthcare goods and services (knowing purchase vs. unknowing purchase).*

Similarly, based on studies on the supply side of the informal economy, this paper seeks to evaluate whether the level of trust that citizens display has any influence on the decision to purchase undeclared healthcare goods and services. Previous literature revealed that both types of trust, namely vertical trust (between the citizens and the government) and horizontal trust (between citizens), are significantly associated with participation on the supply side of the informal economy. As such, when there is a low trust in the government or a low trust that other citizens will behave in a compliant manner, there is a higher likelihood that the individuals will engage themselves in a non-compliant behaviour [29,30]. Indeed, previous research shows how social norms inform the individuals on others‘ behaviour which, in turn shape their own behaviour [31,32,33,34,35]. The limited evidence so far available on the demand-side of the informal economy similarly finds an association between trust and the purchase of undeclared goods and services [6,8]. Thus, we propose to test the following hypotheses:

**Hypothesis 2** **(H2).** *The higher the level of vertical trust the lower is the propensity to buy undeclared healthcare goods and services;*

**Hypothesis 3** **(H3).** *The higher the level of horizontal trust the lower is the propensity to buy undeclared healthcare goods and services.*

To ensure a nuanced understanding of the effect of trust on the purchase of healthcare goods and services, we test these hypotheses in turn for all healthcare purchases from the informal economy, for knowing purchases and for unknowing purchases. In order to do so, we here selected as a case study the four countries in the European Union where this practice is the most prevalent and, as such, enables valid statistical analysis.

The next section will present the materials and methods employed for testing the proposed hypotheses. This section is followed by a presentation of the findings. Finally, the paper discusses the findings and advances the conclusions.

## 2. Materials and Methods

To test the validity of the proposed hypotheses, we here investigate the purchase of undeclared healthcare goods and services in four countries in Southern Europe, namely Cyprus, Greece, Italy and Malta. These countries are member states of the European Union and were selected based on the high prevalence of informal purchases of healthcare services. Indeed, 13% of citizens in Malta, 9% in Cyprus, 7% in Greece and 2% in Italy declared that they have paid for health services which were not registered for tax purposes in the last 12 months prior to survey, making the selected countries included in the analysis the countries with the highest rates of purchase of undeclared healthcare goods and services amongst European member states. Therefore, the high number of positive outcomes in the selected countries (i.e., individuals declaring that they paid for undeclared healthcare goods and services) enabled the statistical data analysis. Data used were extracted from Special Eurobarometer no. 498 on undeclared work, as part of Eurobarometer Wave 92.1 [36]. The fieldwork conducted in September 2019 involved 3048 face-to-face interviews in Cyprus, Greece, Italy and Malta, with individuals over 15 years old. In each of the four countries selected for analytical purposes, a random-probability multi-stage sample design was employed. Sampling points were drawn to cover the analysed territories at NUTS II level (Eurostat’s Nomenclature of Territorial Units for Statistics). The sample in each country matches the universe of the population in terms of region of residence, urbanization, gender and age (for details about the data used, see [37]).

Although the Special Eurobarometer survey no. 498 covered various issues on the supply and demand side of undeclared work, we here confine the discussion to the purchase of undeclared healthcare goods and services. A dummy variable was created with coded value 1 for those citizens who declared that they had paid for undeclared healthcare goods and services (e.g., because there was no Value Added Tax (VAT) receipt, no invoice) and value 0 for those who had not paid for such services in the last 12 months prior to the survey. Moreover, based on the motives of purchasing healthcare services undeclared instead of purchasing them from the regular market, two dummy variables were similarly created to evaluate those citizens buying these services knowing or unknowing. As such, a dummy variable with coded value 1 was created if individuals purchased undeclared healthcare goods and services knowingly, and a dummy variable with coded value 0 was created for those individuals who purchased unknowingly undeclared healthcare goods and services.

To evaluate whether there is an association between vertical trust and the propensity to purchase undeclared healthcare goods and services, a composite index measuring the level of tax morale among citizens is here constructed. Indeed, vertical trust or trust in public authorities is frequently associated with tax morale (for a review on the matter see [38]), the latter being used as a proxy for evaluating vertical trust [29,39]. The tax morale index is here constructed by evaluating the acceptability of five tax evasion behaviours on a scale between 1 and 10. The questions in the survey used value 1 for totally unacceptable and 10 for totally acceptable. However, when computing the index, for easier interpreting the findings, we have used a reverse scale. The higher the index value the greater the tax morale, the higher the vertical trust (trust in authorities).

To evaluate whether there is an association between the horizontal trust and the propensity to purchase undeclared healthcare goods and services we here used the perceived estimated share of the population engaged in undeclared activities in the analysed country. Similar to other studies investigating informal economy [8,30,40,41,42], corruption, informal payments and fraud in healthcare [13,14,19,20,43,44,45,46], we also used individual-level control variables related to the socio-demographic profile of the patient buying undeclared healthcare goods and services (e.g., gender, age, education, household size, employment status, financial status and country of residence).

Considering the dichotomous variables used as dependent variables, we employed logistic regressions. While sampling weights were used for the descriptive statistics as recommended in the wider literature [47,48] and in the Eurobarometer technical specifications [37], for the logistic regressions various estimations with and without the sampling weighting scheme were computed, reflecting the debate in the literature over using such weights in the multivariate analyses [47]. As such, we firstly conducted a logistic regression analysis without sampling weights for all three dependent variables and secondly, we conducted a logistic regression analysis with sampling weights. Third and finally, using multiple imputations missing values were replaced and a logistic regression analysis is conducted by using imputed data. All these estimations were performed to further test and to show the robustness of the findings obtained. In addition, to provide a visual view and help interpret the findings, marginal effects and adjusted predicted probability of purchasing undeclared healthcare goods and services by the level of vertical and horizontal trust are displayed. All findings are reported in the next section.

## 3. Results

Starting with an overview of the tendency to purchase undeclared healthcare goods and services, Table 1 reveals the share of individuals doing so in the 12 months prior to the survey and their motives. As such, in 2019, 13% of citizens in Malta, 9% in Cyprus, 7% in Greece and 2% in Italy reported that they paid for undeclared healthcare goods and services. However, these figures should be treated as lower bound estimates, since we report the results as a percentage of the total population. This is because only some citizens needed healthcare services in the last 12 months prior to the survey and therefore could have paid for this kind of service. For instance, in a study reporting the results of a Eurobarometer survey on the use of healthcare services, out of 27,786 respondents at European Union level, only 21,121 had visited a physician or medical institution in the past 12 months [19]. As such, the share of patients paying for undeclared healthcare goods and services will be larger.

Turning to the motives for buying these undeclared services, Table 1 also reveals the share of those who do this knowingly or unknowingly (multiple answers were possible). Therefore, out of those buying undeclared healthcare goods and services in Greece, 80% did so knowingly and only 9% unknowingly. Similarly, in Cyprus, 65% did so knowingly and only 20% unknowingly. In sharp contrast, only 41% of those buying undeclared healthcare goods and services in Malta did so knowingly and 67% unknowingly.

Exploring the reasons to knowingly pay for undeclared healthcare goods and services, the finding is that in the four analysed countries, the most frequently invoked reasons are the lower price and doing a favour for someone or helping someone in need of money. As such, Table 1 reveals that when paying knowingly for undeclared healthcare goods and services, a lower price is the main reason for doing so (reason cited by 69% of those buying these kinds of services in Greece, 57% in Cyprus, 50% in Italy and 33% in Malta), followed by social ends (42% in Italy, 41% in Cyprus, 31% in Greece and 5% in Malta). This rarely happens because the healthcare service is unavailable or difficult to find on the regular market.

To evaluate whether these undeclared healthcare goods and services are bought more often from private persons or healthcare institutions (public or private), Table 2 reports the variations by the provider of this service. This reveals that overall, undeclared healthcare goods and services are bought from private persons (other than those close to the patient (71% of those buying undeclared healthcare goods and services), firms or businesses in the health sector (35%), known private persons (e.g., friends, neighbours, colleagues, relatives; 27%) and public service providers (16%). However, no matter the provider of the undeclared service, this involves major implications for public health. The use of public office for private gain (i.e., corruption), fiscal fraud by firms or businesses in the health sector (including employee fraud) and undeclared work or fiscal fraud by private persons all negatively affect the resource allocation in healthcare (inefficient use of resources), healthcare access and quality, patient rights and protection [49,50,51,52].

There are also differences in terms of the provider of undeclared services according to whether the acquisition process is knowing or unknowing. As such, according to data in Table 2, those buying knowingly undeclared healthcare goods and services did so more often from a private person they do not know (75%), a known private person (37%) or from a firm or business (28%). Analysing the reason for unknowingly buying undeclared healthcare goods and services, those buying for a lower price did so more often from a private person they do not know (73%), a known private person 41%) or from a firm or business (31%). Similarly, and as Table 2 reveals, those buying unknowingly undeclared healthcare goods and services did so more often from a private person they do not know (66%), a firm or business (54%) or from public service providers (21%).

In order to evaluate the relationship between the propensity to buy undeclared healthcare goods and services and vertical and horizontal trust, Table 3 firstly displays the socio-economic profile of the individuals more likely to buy such services. The results of the logistic regressions evaluating undeclared healthcare goods and services overall (Model 1 and Model 2), knowingly bought (Model 3) or unknowingly bought (Model 4) show that no relationship could be identified with the socio-economic variables. No socio-economic profiles of those buying undeclared healthcare goods and services (knowingly or unknowingly) could be identified, meaning that buyers of this type of service could be found in each socio-economic group. This is the case when solely the socio-economic variables are included in the model evaluating undeclared healthcare goods and services without taking other variables into account (Model 1), when vertical and horizontal trust variables are added to the socio-economic variables while evaluating undeclared healthcare goods and services irrespective of the reason of purchase (Model 2), or when broken down by knowing reasons (Model 3) or unknowing reasons (Model 4) for buying such services (rejecting the Hypothesis H1). As such, one cannot assert that this practice is more common in some socio-economic groups than others even if previous studies identified a relationship between the employment status and citizens self-assessed health [53]. However, this is not the case when evaluating the relationship between the propensity to buy undeclared healthcare goods and services and the country of residence or the vertical and horizontal trust. Indeed, citizens in Malta, Cyprus and Greece are more likely to (knowingly) buy undeclared healthcare goods and services than those in Italy (Models 2 and 3 in Table 3), while only citizens in Malta are more likely to unknowingly buy undeclared healthcare goods and services than those in Italy (Model 4 in Table 3).

By analysing the relationship between undeclared healthcare goods and services and vertical trust (Model 2), a statistically significant negative association is revealed. The higher the vertical trust (measured using tax morale), the lower is the propensity to buy undeclared healthcare goods and services (confirming Hypothesis H2). There is also a strong association between individuals buying undeclared healthcare goods and services and their perceived estimation regarding the share of the population engaged in undeclared activities (horizontal trust or trust in other citizens). Those perceiving a larger share of citizens to be engaged in undeclared activities (i.e., perceiving it to be a common practice in their country) are more likely to buy themselves undeclared healthcare goods and services. This therefore confirms Hypothesis H3. Both H2 and H3 are further confirmed when analysing the propensity to buy undeclared healthcare goods and services as a knowing activity (Model 3). With larger coefficients, a strong association is revealed between individuals knowingly buying undeclared healthcare goods and services and their level of vertical and horizontal trust. Moreover, when patients consider that the estimated share of the population engaged in undeclared activities is large (i.e., over 40%) it is more likely (higher coefficients) that they, themselves, will (knowingly) buy undeclared healthcare goods and services.

Even if vertical and horizontal trust should only play, by chance, a role in explaining the unknowing acquisition of undeclared healthcare goods and services, Model 4 in Table 3 tests if this is the case. As expected, no statistically significant association was identified in respect to vertical trust. By contrast, the perceived estimated share of the population engaged in undeclared activities was identified as associated with unknowing purchases of undeclared healthcare goods and services. However, with the findings in Table 3, one cannot conclude that the higher the perceived estimated share of the population engaged in undeclared activities, the higher is the likelihood to unknowingly buy undeclared healthcare goods and services (i.e., the relationship is significant only for one level, namely the perceived level of spread of 10–40%). Thus, even if effective in dealing with knowingly buying undeclared healthcare goods and services, improving the levels of both vertical and horizontal trust will not reduce the practice of unknowingly buying undeclared healthcare goods and services. Other policy measures are needed in this regard, such as to encourage proactive behaviour in asking for an invoice.

These results on vertical and horizontal trust are further supported by a robustness analysis provided in Table 4. This reveals that the results are broadly the same for all models (Model 2 to 4) regardless of if the logistic regressions are estimated with or without sample weights or with imputed missing data.

**Figure 1:**
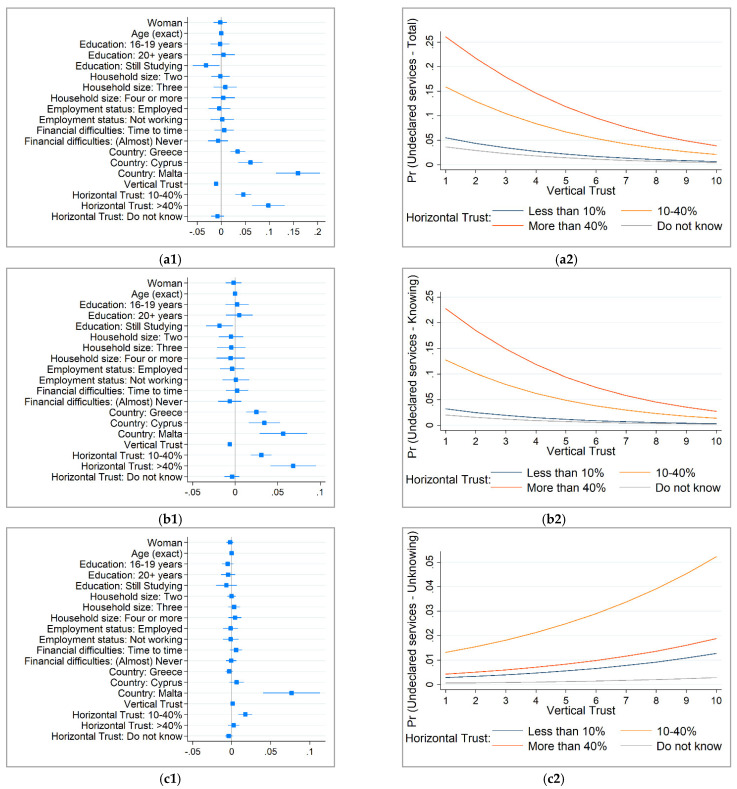
Marginal effects and adjusted predicted probability to buy undeclared healthcare goods and services (after logistic regression). (**a1**) Undeclared healthcare goods and services: Total (Marginal effects); (**a2**) Undeclared healthcare goods and services: Total (Predicted probability), by Vertical and Horizontal Trust); (**b1**) Undeclared healthcare goods and services: Knowing (Marginal effects); (**b2**) Undeclared healthcare goods and services: Knowing (Predicted probability), by Vertical and Horizontal Trust); (**c1**) Undeclared healthcare goods and services: Unknowing (Marginal effects); (**c2**) Undeclared healthcare goods and services: Unknowing (Predicted probability), by Vertical and Horizontal Trust). *Source*: author‘s estimations based on data extracted from Special Eurobarometer 498. Notes: predicted probability for a ‘representative’ individual computed with the mean and mode of the socio-demographic variables: 49 years old woman, 16–19 years old when stopped education, living in Italy in a 2 persons household, not working and facing financial difficulties from time to time.

To graphically display the magnitude of the effect of each variable included in the analysis on the likelihood to purchase (knowingly and unknowingly) undeclared healthcare goods and services, Figure 1(a1–c1) displays the marginal effects and Figure 1(a2–c2) the adjusted predicted probability by vertical and horizontal trust.

According to Figure 1(a1,b1), the country of residence, vertical and horizontal trust have the largest effect in explaining the prevalence of (knowingly) undeclared healthcare goods and services. Figure 1(a2,b2) further investigate the role of vertical and horizontal trust in explaining the decision to (knowingly) buy undeclared healthcare goods and services by computing the predicted probability by these two variables. When having a low level of vertical trust, the probability to (knowingly) purchase undeclared healthcare goods and services is much higher than when having a high level of vertical trust. Similarly, horizontal trust plays a central role in predicting (knowingly) undeclared healthcare goods and services. As such, with low levels of horizontal trust (high perceived estimated percent of the population engaged in undeclared activities), the probability to (knowingly) buy undeclared healthcare goods and services is much higher. Moreover, Figure 1(a2,b2) clearly display how improving either the level of vertical trust, either the level of horizontal trust will significantly reduce the prevalence of (knowingly) undeclared healthcare goods and services. Indeed, maximum results could be obtained with high levels of both vertical and horizontal trust.

Marginal effects and predicted probabilities by vertical and horizontal trust for those unknowingly buying undeclared healthcare goods and services are displayed in Figure 1(c1,c2). Beside country of residence, both figures reveal a single statistically significant association between unknowingly undeclared healthcare goods and services and those considering that the share of population engaged in undeclared activities is between 10 and 40%. However, according to Figure 1(c1,c2), its effect on the prevalence of unknowingly undeclared healthcare goods and services is very small.

## 4. Discussion

This paper has explored the purchase of healthcare undeclared goods and services in four member states of the European Union from Southern Europe, namely Cyprus, Greece, Italy and Malta. The finding is that 13% of citizens in Malta, 9% in Cyprus, 7% in Greece and 2% in Italy purchased undeclared goods and services from healthcare sector in the past 12 months prior to survey. However, considering that not all the respondents to the survey needed healthcare or visited a hospital or a clinic in the year prior to survey, these figures need to be seen as lower bound estimates. Variations exist when investigating whether the purchase was knowing or unknowing (i.e., the purchase realizing that they have made a purchase from the informal economy only afterwards, because they have not received an invoice or a receipt). Out of those buying undeclared healthcare goods and services in Greece, 80% did so knowingly and only 9% unknowingly. Similarly, in Cyprus, 65% did so knowingly and only 20% unknowingly. In sharp contrast, only 41% of those buying undeclared healthcare goods and services in Malta did so knowingly and 67% unknowingly. Undeclared healthcare goods and services are bought more frequently from private persons (other than those close to the patient; 71% of those buying undeclared healthcare goods and services), firms or businesses in the health sector (35%), known private persons (e.g., friends, neighbours, colleagues, relatives; 27%) and public service providers (16%). However, those buying unknowingly undeclared healthcare goods and services did so more often from public service providers (21%) than those making a knowing decision, displaying the fact that using the public office for private gain by the medical staff is rather extensive. However, no significant differences between the socio-demographic characteristics of those purchasing healthcare undeclared goods and services on a knowing basis and those making such purchases on an unknowing basis have been identified. As such, the finding is that when analysing the demand-side of the informal economy there is no similar dual informal labour market divide, as is the case for the supply side of the informal economy.

Nevertheless, a strong association between the likelihood to purchase healthcare undeclared goods and services and the level of trust (in government and between citizens) displayed by citizens has been identified. Important to mention however is that this association has been identified only for knowing purchases from the informal economy. This has important policy implications for tackling this phenomenon which negatively impact the resource allocation in healthcare, healthcare access and quality and patient rights and protection. As such, different policy approaches are required to address the two groups of purchasers. Those who unknowingly made such purchases should be encouraged to act legitimately using policy initiatives that encourage them to ask for a receipt for all services. Such examples include, for instance, receipts lotteries or service vouchers [54,55]. Indeed, considering that 71% of those purchasing undeclared healthcare goods and services made these purchases from private persons, other than those close to the patient, a scheme of vouchers that would allow them to employ private persons for specific goods or services could be effective in reducing the prevalence of this practice. Other effective policies might also include the introduction of certified cash registers across all health services accompanied by adverts and posters informing citizens that unless they receive a printed receipt, they are not obliged to pay for any bills received and can make a complaint at a certain hyperlink. This would require the development of a complaint reporting tool by the national health services where requests for informal payments can be reported. Meanwhile, for those who knowingly make such purchases, a wider range of policy initiatives are necessary. Firstly, regardless of the reasons that made them purchase undeclared healthcare goods and services (i.e., lower price, faster or better service, social ends or lack of availability on the formal market), an array of policies aimed at fostering trust are necessary. On the one hand, this can entail governments using education and awareness raising campaigns which highlight the benefits of operating in the formal economy. On the other hand, however, this can also entail governments nurturing the trust of citizens by improving the quality of governance [56,57], procedural justice [58,59], procedural fairness [60] and redistributive justice [61]. Third and finally, this article reveals the need to improve the horizontal trust of citizens that other citizens operate in a compliant manner and do not buy undeclared goods and services. This might include not publicizing the high levels of non-compliance, which reduces horizontal trust, and instead targets communications to citizens on the very high level of overall compliance, both of their peer group (e.g., in their sector, locality) and the wider society [62].

## 5. Conclusions

This paper is the first study to investigate how both types of trust (i.e., between citizens and the government and between the citizens) influence the purchase of undeclared goods and services, taking as a case study the healthcare sector. The findings show that trust is significantly associated with the greater likelihood to purchase undeclared healthcare goods and services if the decision to make a purchase from the informal economy is knowing. For the unknowing purchases, no straightforward relationship has been identified. This paper was unable, however, to look deeper at the link between the purchase of undeclared healthcare goods and services and other informal practices prevalent in the healthcare sector, such as the informal payments made by patients to the healthcare practitioners. It would be important to explore, especially for the unknowing purchases, whether the use of public office for private gain (i.e., selling healthcare goods and services without registering them) is related or not with the informal payments. It might be the case that, the same medical staff require an informal payment for providing goods or services to the patients in need, which is also undeclared, being as such involved in multiple illegal activities. This could involve qualitative research to explore in greater depth the reasons and motives of patients and medical staff involved in the purchase of undeclared goods and services.

Furthermore, this study is conducted on a narrow range of Southern European countries. Whether similar findings are identified when a wider range of countries and global regions are evaluated now needs further research. The current methodology could be replicated in these other countries and global regions to evaluate whether this is the case. Whether these findings are valid when other sectors or other regions are investigated needs to be explored in further studies.

If this study now encourages further studies in a wider range of countries and global regions, then it will have fulfilled one of its intentions. If it also encourages governments in general, and health service authorities more particularly, to start to experiment with the above suggested policy initiatives to improve vertical and horizontal trust, then it will have fulfilled its wider intention.

## Figures and Tables

**Table 1:** Undeclared healthcare goods and services by buying reasons and country (%; *n* = 2953).

	Undeclared Healthcare Goods and Services						
	Total	*Of Which ^(^*^1)^:					
		Knowing					Unknowing
		All Knowing Reasons	By Reason:				
			Lower Price	Faster/ Better Service	Social Ends	Unavailable ^(2)^	
Cyprus	9	65	57	41	41	16	20
Greece	7	80	69	24	31	16	9
Italy	2	58	50	8	42	8	46
Malta	13	41	33	4	5	3	67

*Notes: ^(^*^1)^ multiple answers possible; other reason, refusal, and do not know are not displayed; ^(2)^ service unavailable/difficult to find on regular market. Source: author’s estimations based on data extracted from Special Eurobarometer 498.

**Table 2:** Undeclared healthcare goods and services by provider (%; *n* = 209).

		Provider of Undeclared Services ^(1)^			
		Known Private Persons	Other Private Persons	Firms or Businesses	Public Service Providers
**Undeclared Healthcare Goods and Services**	**Total**	27	71	35	16
	**Of which** ^(**1**)^ **:**				
	**Knowing**	37	75	28	16
	Lower price	41	73	31	17
	Faster/Better	62	52	54	43
	Social ends	37	77	19	11
	Unavailable ^(2)^	38	75	49	35
	**Unknowing**	3	66	54	21

*Notes:*^(1)^ multiple answers possible; other reason/provider, refusal and do not know are not displayed; ^(2)^ service unavailable/difficult to find on regular market. Source: author’s estimations based on data extracted from Special Eurobarometer 498.

**Table 3:** Logistic regression of the propensity to buy undeclared healthcare goods and services in Cyprus, Greece, Italy and Malta.

	Model 1 Undeclared Healthcare Goods and Services			Model 2 Undeclared Healthcare Goods and Services			Model 3 Undeclared Healthcare Goods and Services			Model 4 Undeclared Healthcare Goods and Services		
	*Total*			*Total*			*Knowing*			*Unknowing*		
	Coef.		(SE)	Coef.		(SE)	Coef.		(SE)	Coef.		(SE)
***Socio-*** ***economic*** ***profile (control variables)***												
*Gender* (R: Man)												
Woman	−0.207		(0.151)	−0.047		(0.157)	−0.076		(0.196)	−0.265		(0.266)
*Age* (exact)	0.002		(0.006)	0.004		(0.006)	−0.006		(0.008)	0.013		(0.012)
*Education* (R: Up to 15 years old when stopped full time education)												
16–19	0.007		(0.209)	−0.050		(0.217)	0.093		(0.283)	−0.531		(0.340)
20+	0.184		(0.243)	0.100		(0.249)	0.189		(0.315)	−0.467		(0.442)
Still Studying ^(1)^	−1.064	*	(0.622)	−1.047		(0.640)	−1.422	*	(0.861)	−0.773		(0.975)
*Household composition, aged 15+* (R: One person)												
Two	−0.003		(0.219)	−0.036		(0.227)	−0.194		(0.271)	−0.024		(0.383)
Three	0.228		(0.257)	0.180		(0.265)	−0.176		(0.328)	0.360		(0.448)
Four or more	0.273		(0.265)	0.088		(0.274)	−0.212		(0.331)	0.462		(0.461)
*Employment status* (R: Self-employed)												
Employed	−0.260		(0.248)	−0.085		(0.259)	−0.154		(0.301)	−0.168		(0.532)
Not working	−0.076		(0.261)	0.047		(0.275)	0.033		(0.321)	−0.140		(0.556)
*Financial difficulties* ^(2)^ (R: Most of the time)												
From time to time	0.033		(0.211)	0.132		(0.218)	0.085		(0.254)	0.604		(0.487)
Almost never/Never	−0.165		(0.236)	−0.158		(0.243)	−0.277		(0.294)	−0.107		(0.511)
*Country* (R: Italy)												
Greece	1.041	***	(0.250)	1.166	***	(0.256)	1.420	***	(0.320)	−0.729		(0.528)
Cyprus	1.345	***	(0.264)	1.615	***	(0.272)	1.675	***	(0.346)	0.702		(0.468)
Malta	1.834	***	(0.255)	2.550	***	(0.275)	2.121	***	(0.366)	2.664	***	(0.389)
***Vertical and Horizontal Trust***												
*Vertical Trust* (Tax morale)				−0.242	***	(0.038)	−0.261	***	(0.044)	0.171		(0.121)
*Horizontal Trust* (estimated % of the population engaged in undeclared activities (R: Less than 10%))												
10–40%				1.179	***	(0.265)	1.484	***	(0.382)	1.575	***	(0.482)
Over 40%				1.806	***	(0.289)	2.188	***	(0.401)	0.414		(0.637)
Do not know				−0.423		(0.389)	−0.482		(0.624)	−1.541	*	(0.854)
Constant	−3.561	***	(0.554)	−2.865	***	(0.656)	−2.781	***	(0.812)	−7.542	***	(1.533)
Observations	2882			2775			2775			2775		
Log likelihood	−705.16			−637.12			−442.96			−254.26		
χ^2^	78.73			198.54			139.49			173.89		
*p*>	0.000			0.000			0.000			0.000		

*Notes*: ^(1)^ and no full-time education; ^(2)^ in paying household bills; *** *p* < 0.01, ** *p* < 0.05, * *p* < 0.1; SE = Standard Errors (in parentheses). *Source*: author’s estimations based on data extracted from Special Eurobarometer 498.

**Table 4:** Logistic regression—robustness.

		Socio-Demographic Control Variables	*Vertical and Horizontal Trust*				*n*	Prob. > χ^2^/F
			Vertical Trust (Tax Morale)	Horizontal Trust (Estimated % Engaged in Undeclared Activities (R: Less Than 10%)				
				10–40%	Over 40%	DK ^(2)^		
Model 2 Undeclared Healthcare Goods and Services (Total)	No sample weights	Included	−0.242 *** (0.038)	1.179 *** (0.265)	1.806 *** (0.289)	−0.423 (0.389)	2775	0.000
	With sample weights	Included	−0.155 *** (0.059)	1.710 *** (0.469)	1.995 *** (0.501)	0.346 (0.846)	2775	0.000
	Imputed missing data ^(1)^	Included	−0.238 *** (0.038)	1.200 *** (0.265)	1.811 *** (0.289)	−0.629 (0.387)	3048	0.000
Model 3 Undeclared Healthcare Goods and Services (Knowing)	No sample weights	Included	−0.261 *** (0.044)	1.484 *** (0.382)	2.188 *** (0.401)	−0.482 (0.624)	2775	0.000
	With sample weights	Included	−0.279 *** (0.064)	1.876 *** (0.685)	2.166 *** (0.702)	−0.847 (1.005)	2775	0.000
	Imputed missing data ^(1)^	Included	−0.255 *** (0.044)	1.491 *** (0.388)	2.174 *** (0.411)	−0.648 (0.626)	3048	0.000
Model 4 Undeclared Healthcare Goods and Services (Unknowing)	No sample weights	Included	0.171 (0.121)	1.575 *** (0.482)	0.414 (0.637)	−1.541 * (0.854)	2775	0.000
	With sample weights	Included	0.181 (0.147)	3.332 *** (0.545)	2.443 ** (0.957)	−1.793 ** (0.881)	2775	0.000
	Imputed missing data ^(1)^	Included	0.162 (0.122)	1.596 *** (0.483)	0.409 (0.636)	−1.718 ** (0.841)	3048	0.000

*Notes*: ^(1)^ Multiple Imputations for data missing at random, Incomplete–Imputed: Undeclared healthcare goods and services (Total)—95, Undeclared healthcare goods and services (Knowing)—95, Undeclared healthcare goods and services (Unknowing)—95, Education—41, Financial difficulties—45, Vertical Trust—104, Horizontal Trust—50; ^(2)^ DK—Do not know; *** *p* < 0.01, ** *p* < 0.05, * *p* < 0.1; Standard Errors in parentheses. Source: author’s estimations based on data extracted from Special Eurobarometer 498.

## Data Availability

Publicly available dataset used in this study; European Commission, Brussels (2020). Eurobarometer 92.1 (2019). Kantar Public [producer]. GESIS Data Archive, Cologne. ZA7579 Data file Version 1.0.0. Available at: https://doi.org/10.4232/1.13432 (accessed on 12 June 2021).
